# The Potential Therapeutic Effects of Tadalafil on the Endothelium in a Subarachnoid Hemorrhage Animal Model: Insights from Immunohistochemical Staining

**DOI:** 10.3390/cimb46090567

**Published:** 2024-08-29

**Authors:** Kwang Hyon Park, Hyon-Jo Kwon, Eun-Oh Jeong, Hyeon-Song Koh, Jeongwook Lim

**Affiliations:** 1 Department of Neurosurgery, Chungnam National University Sejong Hospital, Sejong 30099, Republic of Korea; tooez4me@naver.com; 2 Department of Neurosurgery, Chungnam National University School of Medicine, Daejeon 305764, Republic of Korea; solesoul@daum.net (H.-J.K.); kohhs@cnu.ac.kr (H.-S.K.); 3 Department of Neurosurgery, Chungnam National University Hospital, Daejeon 34134, Republic of Korea; jeo5555@daum.net

**Keywords:** aneurysm, subarachnoid hemorrhage, delayed cerebral ischemia, nimodipine, tadalafil, endothelium, apoptosis

## Abstract

This study investigated the potential of phosphodiesterase type 5 (PDE-5) inhibitors, specifically tadalafil, in preventing the delayed cerebral ischemia (DCI) post-rupture of cerebral aneurysms. A total of 19 rabbits were used in this study, divided into different treatment groups, including nimodipine alone, tadalafil alone, and a combination of nimodipine and tadalafil. Both nimodipine and tadalafil showed some impact on reducing endothelial apoptosis in the basilar arteries, although the effects were not statistically significant. Notably, the nimodipine group exhibited significantly lower levels of Bax in the small arterioles compared to the SAH group. These findings suggest that while tadalafil may not directly prevent endothelial cell death like nimodipine, its neuroprotective properties hint at its potential utility in DCI treatment. Further research involving a broader range of apoptosis-related proteins is recommended to enhance our understanding in this area.

## 1. Introduction

Delayed cerebral ischemia (DCI) is a critical complication after cerebral aneurysm rupture. DCI can occur between 3 and 14 days after the rupture of a cerebral aneurysm, excluding secondary contributing causes such as rebleeding, hydrocephalus, infection, and systemic complications, and has some overlap with the pathophysiological mechanisms of early brain injury [1]. The mechanisms underlying DCI include cerebral vasospasm, microcirculatory disturbances, microthrombosis, neuroinflammation, capillary transit time heterogeneity, and cortical spreading depolarization [2,3]. One option for reducing DCI risk is oral nimodipine [4]. It improves the microcirculation by decreasing thromboxane A2 secretion from platelets [5], activates fibrinolysis to inhibit microthrombus formation [6], and prevents vascular responses to cortical spreading depression in ischemic areas [7]. In addition, Cho et al. observed endothelial apoptosis in a vasoconstricted basilar arterial wall in an SAH animal model and argued that it could be related to DCI, and that nimodipine could prevent DCI by maintaining endothelial integrity and vasoactive amine balance [8]. In addition to nimodipine, various drugs are being investigated, such as other calcium channel blockers, endothelin receptor antagonists, statins, and Rho-kinase inhibitors [2]. Although phosphodiesterase (PDE) inhibitors are medically used for intra-arterial infusion in refractory cerebral vasospasm [9,10], similar phosphodiesterase type 5 (PDE-5) inhibitors have been reported to have therapeutic potential in DCI [11,12,13,14].

PDE inhibitors, including papaverine and milrinone, have been applied intra-arterially in medically refractory cerebral vasospasm patients [9,10], while cilostazol, a PDE type 3 inhibitor, is also used as a treatment for DCI because it inhibits platelet aggregation and dilates the cerebral vasculature [15]. Originally used for erectile dysfunction and pulmonary hypertension, PDE-5 inhibitors increase cyclic guanosine monophosphate (cGMP) levels and intracellular nitric oxide (NO), leading to vasodilation [16]. Recently, the effects of PDE-5 inhibitors on the cerebral vasculature have been reported. Elevated expression of PDE-5 has been observed in an animal model of SAH [11]. In addition, PDE-5 inhibitors suppress smooth muscle proliferation in the tunica media, resulting in cerebral vessel dilation [13], and restore damaged endothelial integrity, inducing an increase in cerebral blood flow [12], and have been associated with improved cognitive function [14]. Furthermore, PDE-5 inhibitors activate the phosphoinositide 3 kinase/serine-threonine kinase/endothelial NO synthase (PI3K/AKT/eNOS) signaling pathway in the endothelium [17,18]. This evidence suggests that PDE-5 inhibitors could ameliorate DCI, similar to nimodipine. The PDE-5 inhibitor with the longest half-life is tadalafil [14].

Apoptosis is a crucial mechanism for maintaining cellular homeostasis, and various proteins affect this mechanism [19,20]. Members of the B-cell lymphoma 2 (BCL2) protein family are key regulators with pro- (Bax) and anti-apoptotic activities (BCL2) [20]. These regulators are held in a fine, delicate balance in healthy cells. Actually, they can cause cells to irreversibly progress toward cell death or, conversely, allow cells to permanently escape apoptosis and make themselves malignant clones [20,21]. The expression of BCL2 in cerebral ischemia and p53 in cerebral vasospasm models is known [22,23].

We orally administered tadalafil and nimodipine to an SAH animal model and examined the effects of these drugs on apoptosis in the basilar arteries and arterioles using hematoxylin and eosin (H&E), Verhoeff–Van Gieson, and immunohistochemical (IHC) staining of apoptosis-related proteins.

## 2. Materials and Methods

This study was approved by our animal ethics committee, and all experimental procedures were conducted in accordance with the ARRIVE 2.0 guidelines.

### 2.1. Experimental Animal and Grouping

New Zealand white rabbits (2.5–3.0 kg) of both sexes were used. The number of experimental animals was calculated using G*power version 3.1.9.7 (power = 80%, significance level = 0.5, pooled prevalence = 0.82). The experimental animals were randomly divided into four groups after SAH induction: a group administered saline solution (SAH group), a group administered oral nimodipine (nimodipine group), a group administered tadalafil (tadalafil group), and a group administered both nimodipine and tadalafil (combined group).

### 2.2. Induction of SAH in Experimental Animals and Administration of Drugs

All experimental animals were fasted for 6 h before the start of the experiment, and then, a mixture of ketamine (45 mg/kg; Huons Co., Jaecheon, Republic of Korea) and xylazine (5 mg/kg; Bayer AG, Leverkusen, Germany) was intramuscularly injected into the thigh area. After anesthesia, the suboccipital area and the inside of both ears were waxed using depilatory cream, and the animal was fixed to the experimental table in a prone position. The denuded suboccipital area and ears were sterilized using 75% alcohol, and 1% lidocaine (Daihan Pharm Co., Ansan, Republic of Korea) was injected as local anesthesia into the occipitoatlantal area. Under fluoroscopy guidance, the dura was punctured through the midline of the occipitoatlantal area with a 25-gauge butterfly needle. The location of the needle in the cisterna magna was confirmed by observing cerebrospinal fluid through the needle and by fluoroscopy after contrast agent administration through the needle. After slowly draining about 2 mL of cerebrospinal fluid, unclotted arterial blood was collected from the central artery of the ear using a 25-gauge butterfly needle and injected into the cisterna magna through a butterfly needle. Afterward, the experimental animal was maintained in the Trendelenburg position for about 20 min to ensure good diffusion of blood to the basal cistern.

The experimental animals that had all recovered after 3 h were assigned to the SAH, nimodipine, tadalafil, and combined groups through blocked randomization. The SAH group (n = 4) was administered 3 mL physiological saline every 4 h, the nimodipine group (n = 5) was administered nimodipine (18.5 mg/kg/day) in powder form, and the tadalafil group (n = 5) was administered tadalafil (0.128 mg/kg/day) in powder form. In the combined group (n = 5), nimodipine (18.5 mg/kg/day) and tadalafil (0.128 mg/kg/day) were mixed with 3 mL of physiological saline and administered through a gastric tube. Nimodipine was administered at one-sixth of the total dose every 4 h, and tadalafil was administered at half of the total dose every 12 h. The experimental animals with SAH were sacrificed by air embolism after anesthesia using the same anesthesia method on the third day. The occipital area was removed, the suboccipital muscles were dissected, suboccipital craniectomy and C1 laminectomy were performed, the dura was opened, and the brain stem and vertebrobasilar artery system were extracted.

### 2.3. H&E and IHC Staining

In the endothelium of the basilar arteries and small arterioles, the anti-apoptotic protein BCL2 (cat. no. BS-0032R; Thermo Fisher Scientific, Inc., Los Angeles, CA, USA) and the apoptotic protein Bax (cat. No. BS-0127R; Thermo Fisher Scientific, Inc.) were subjected to IHC staining using the avidin–biotin complex method according to the manufacturer’s protocol. Harvested tissues were embedded in 10% formalin and paraffin wax and sectioned to a 5-µm thickness. The resulting tissue microarray sections were stained with H&E, IHC, and Verhoeff–Van Gieson stains. The stained tissue sections were shown to contain the basilar artery in the basilar groove.

The tissue sections were deparaffinized, rehydrated, immersed in an antigen retrieval solution (EDTA, pH 8), blocked in 3% hydrogen peroxide, and washed with phosphate-buffered saline (PBS). Primary antibody was diluted in 3% bovine serum albumin/PBS to approximately 1:500 and applied to the tissue sections: BCL2 for 30 min at room temperature and Bax overnight at 4 °C in a humidified chamber. The slides were washed with PBS and incubated with the appropriate anti-rabbit IgG secondary antibody.

Verhoeff–Van Gieson staining was performed to check for damage to the internal elastic lamina of the basilar artery. The paraffinized tissue block was first deparaffinized and hydrated and then stained with Verhoeff’s solution for 1 h and washed 2 or 3 times with tap water. After differentiating with 2% ferric chloride for about 2 min, the tissue was rinsed with tap water, perfused with 5% sodium thiosulfate for about 1 min, and counterstained with Van Gieson’s solution for about 5 min. The tissue was dehydrated with alcohol and washed twice with xylene.

### 2.4. IHC Analysis and Statistical Analysis

An independent neuropathologist who was blinded to group information analyzed the scanned images using the CaseViewer application (version 2.4, 3DHISTECH Ltd., Budapest, Hungary). Only arterioles less than 50 μm in diameter were included in this study. BCL2 and Bax expressed in the endothelium of the basilar arteries and arterioles were observed, and the expression level of each antibody was analyzed for intensity and continuity using the phenotypic scoring system: 3, strong staining or encircled staining; 2, moderate staining or partially continuous staining; 1, weak staining or scattered staining; 0, absence of brown staining. The intensity of brown color was based on von Luschan’s Chromatic Scale [24]: a score of 14 or less was negative, 15 to 19 was weak, 20 to 26 was moderate, and 27 or more was strong staining. The degree of expression was indicated by summing the scores for each of intensity and continuity.

Statistical analysis was performed using SPSS, version 22.0 (IBM Corp., Armonk, NY, USA). Kruskal–Wallis analysis with Bonferroni correction was used for the comparative analysis of the BCL2 and Bax scores between groups. All tests were two-sided, with 0.05 denoting statistical significance.

## 3. Results

In total, 19 rabbits were included in the experiment. The analysis included 19 basilar arteries (SAH group, n = 4; nimodipine group, n = 5; tadalafil group, n = 5; combined group, n = 5) and 25 arterioles (SAH group, n = 6; nimodipine group, n = 8; tadalafil group, n = 6; combined group, n = 5). All experimental animals in which SAH was induced recovered from the anesthesia 3 h after induction but exhibited reduced movement and food intake. Except for two rabbits in the SAH group, movement returned to almost normal on the third day after SAH induction, but feed intake was still reduced. Two rabbits in the SAH group showed significantly slower movement upon stimulation.

### 3.1. H&E and IHC Staining Findings

**Figure 1:**
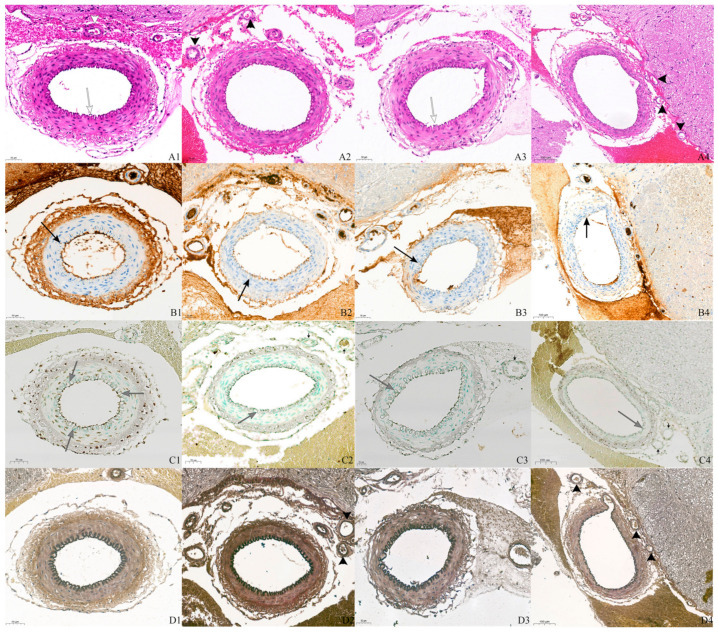
The microscopic results of tissue staining. In hematoxylin and eosin (H&E) and Verhoeff–Van Gieson staining, all groups exhibited a corrugated internal elastic lamina, proliferation of the smooth muscle layer, and SAH around the basilar artery. Endothelial desquamation was evident in the SAH and tadalafil groups (white arrow, **A1**,**A3**). Compared to the other groups, the basilar artery of the combined group showed less smooth muscle proliferation and less wrinkling of the internal elastic lamina (**A4**,**D4**). In the arterioles, the SAH group showed a corrugated internal elastic lamina, smooth muscle proliferation, and a change in the shape of the smooth muscle nucleus due to tonic contracture of the smooth muscle (large white arrowhead: **A1**,**D1**). In the drug-administered groups, wrinkles of the internal elastic lamina and smooth muscle proliferation were not observed (large black arrowhead; **A2**–**A4**,**D2**–**D4**). With immunohistochemical staining of the basilar artery for BCL2 (**B**; large black arrow) and Bax (**C**; large gray arrow), most endothelial cells in all groups were stained, but the degree of staining and continuity of stained endothelial cells were lower in the drug-administered groups (**B2**–**B4**,**C2**–**C4**) than in the SAH group (**B1**,**C1**). With immunohistochemical staining of the arterioles, although there was a difference in the degree of BCL2 expression in the arterioles, stained endothelial cells were observed in both the SAH and drug-administered groups. However, Bax-stained endothelial cells were observed in the SAH (small white arrow, **C1**) group but not in the drug-administered groups (small black arrow, **C2**–**C4**). All slides were imaged at 20× magnification. First row, H&E staining (**A**); second row, immunohistochemical staining of BCL2 (**B**); third row, immunohistochemical staining of Bax (**C**); fourth row, Verhoeff–Van Gieson staining (**D**). First column (**1**), SAH group; second column (**2**), nimodipine group; third column (**3**), tadalafil group; fourth column (**4**), combined group. SAH: subarachnoid hemorrhage.

In the basilar arteries, the findings in all groups included hemorrhage around the basilar artery and smooth muscle proliferation and constriction in the tunica media (Figure 1). A continuous and corrugated internal elastic lamina was evident in all groups with H&E stain and Verhoeff–Van Gieson stain, and there were no differences between groups. However, desquamation of endothelial cells was seen in the SAH and tadalafil groups. In endothelial cells, the expression levels of BCL2 and Bax were lower in the drug-administered groups than in the SAH group, and discontinuity of staining was also more common in the drug-administered groups.

In the small arterioles, the SAH group had a corrugated internal elastic lamina, and its continuity was maintained, but smooth muscle proliferation was not observed. In the drug-administered groups, the internal elastic lamina was relatively wrinkle-free and uninterrupted, and no smooth muscle proliferation was seen. The expression levels of BCL2 and Bax in endothelial cells were lower in the drug-administered groups than in the SAH group.

### 3.2. Phenotypic Scoring of BCL2 and Bax Expression

In the basilar arteries, BCL2 and Bax expression was observed in all groups, but compared with SAH, BCL2 and Bax were expressed at nonsignificantly lower levels in the nimodipine, tadalafil, and combined groups (*p*^BCL2^ = 0.124, *p*^Bax^ = 0.050).

**Figure 2:**
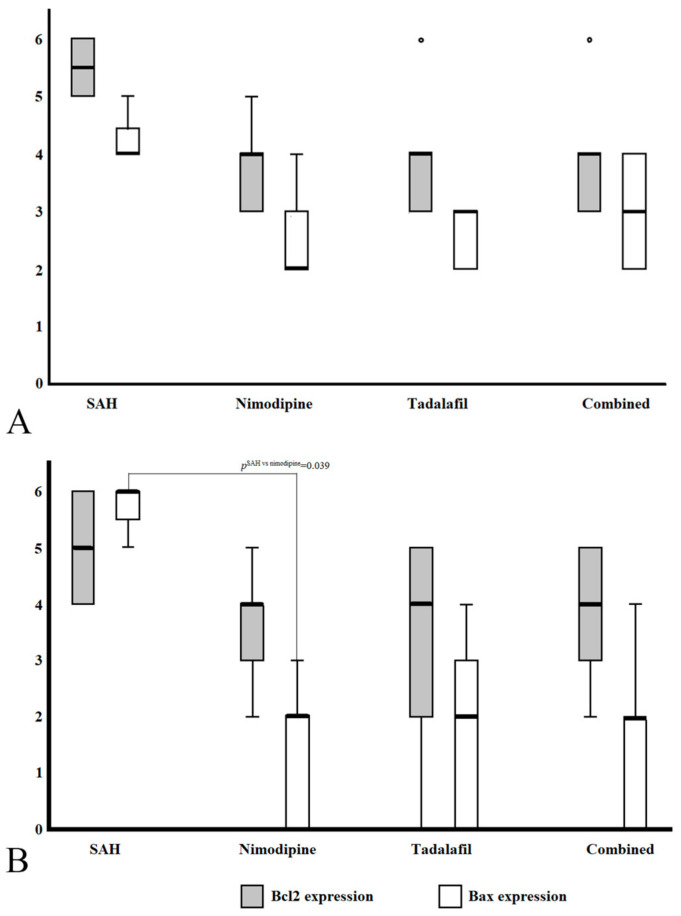
Box plot summary of the expression levels of BCL2 and Bax in the basilar arteries (**A**) and arterioles (**B**). (**A**): BCL2 and Bax in the basilar arteries were nonsignificantly less expressed in the drug-administered groups (nimodipine, tadalafil, and combined groups) compared with the SAH group (*p*^BCL2^* = 0.124, *p*^Bax^* = 0.050). (**B**): In the arterioles, like the basilar artery, the expression of BCL2 was nonsignificantly reduced in the drug-administered groups (*p*^BCL2†^ = 0.420). However, the expression of Bax was significantly lower in the drug-administered group (*p*^Bax†^ = 0.022). Finally, in the comparison among groups, the nimodipine group was significantly different from the SAH group (*p*^SAH vs. nimodipine^ = 0.039) and nonsignificantly different from the tadalafil and combined groups (*p*^SAH-tadalafil^ = 0.092, *p*^SAH vs. combined^ = 0.054). Analyses were conducted using the Kruskal–Wallis test, and the *p* values were corrected using Bonferroni correction. *: *p* value of basilar arteries, ^†^: *p* value of arterioles.

In the arterioles, BCL2 was expressed in all groups and, like the basilar arteries, exhibited nonsignificantly lower expression in all drug-administered groups compared with the SAH group (*p* = 0.349). In contrast, Bax expression showed a significantly unequal distribution across groups (*p* = 0.022). In the groupwise comparison of Bax, its expression was significantly lower compared with SAH in all groups administered the drug (*p*^SAH vs. nimodipine^ = 0.06, *p*^SAH-tadalafil^ = 0.015, *p*^SAH vs. combined =^ 0.09, *p*^Nimodipine vs. tadalafil^ = 0.751, *p*^Nimodipine vs. combined^ = 0.908, *p*^Tadalafil vs. combined^ = 0.840). However, after Bonferroni correction, the result was significant only for a comparison of the group administered nimodipine and the SAH group (*p*^SAH vs. nimodipine^ = 0.039, *p*^SAH-tadalafil^ = 0.092, *p*^SAH vs. combined^ = 0.054). Table 1 summarizes the comparative analysis of the immunohistochemical staining of BCL2 and Bax in the basilar arteries and penetrated arterioles. Figure 2 shows the degree of expression of BCL2 and Bax in the basilar arteries and arterioles as a box plot.

## 4. Discussion

DCI is the most severe complication following a ruptured cerebral aneurysm and exhibits a prevalence of about 30% [18]. The development of various neurovascular surgical techniques and devices has lowered the rate of immediate mortality after rupture but has not yet lowered the morbidity and mortality of DCI [1]. The outcome of DCI is unaffected by the resolution of cerebral vasospasm, one of the pathological mechanisms of DCI [17,25]. This is due to the multifactorial pathophysiology of DCI and because it cannot be solved with simple treatment methods. However, oral nimodipine is a well-established drug that can ameliorate DCI through a complex process.

Early brain injury that occurs within 72 h after a ruptured cerebral aneurysm is caused by transient global cerebral ischemia, cerebral edema, and intracerebral hemorrhage [26], and DCI that occurs after 72 h is caused by cerebral vasospasm, microvascular disturbance, microthrombosis, cortical spreading ischemia, capillary transit time heterogeneity, and neuroinflammation [1,2,3,26]. However, the mechanisms of the two types of brain damage overlap and include inflammation, increased endothelin, decreased NO, oxidative stress, blood–brain barrier breakdown, and cellular apoptosis [2,27]. In particular, endothelial apoptosis is observed in SAH-induced experimental animals [26], and oral nimodipine has a preventive effect on endothelial apoptosis in penetrated arterioles [8]. Endothelium homeostasis maintains the balance of vasoactive amines and has a significant impact on microcirculatory disturbance, blood–brain barrier breakdown, and microthrombosis, while damage to the endothelium causes smooth muscle cell proliferation and neuroinflammation [26].

Many attempts have been made to prevent DCI. In addition to oral nimodipine (representative calcium channel blocker), clazosentan (endothelin antagonist), tirilazad (nonglucocorticoid 21-amniosteroid), fasudil (Rho-kinase inhibitor), and cilostazol (PDE type 3 inhibitor) have been tried [2,4,17,25]. However, except for oral nimodipine, these drugs do not yet have sufficient evidence supporting their use or are used only in a limited number of countries [2].

Papaverine, milrinone, and cilostazol, which have already been applied for cerebral vasospasm and DCI, are PDE inhibitors. Papaverine and milrinone vasodilate constricted cerebral arteries via intra-arterial infusion in medically refractory cerebral vasospasm, and cilostazol has been used for cerebral vasospasm and DCI because it inhibits platelet aggregation and dilates cerebral vessels [9,10,15]. PDE-5 inhibitors, which have been used to treat erectile dysfunction and pulmonary hypertension, have been reported in animal experiments to effectively prevent cerebral ischemia [11,14,28]. In an animal model of SAH and a systemic review of a human study, the administration of tadalafil inhibited smooth muscle cell proliferation and vasodilated the cerebral vasculature, an effect similar to the neuroprotective function of oral nimodipine [12,13]. In ischemic animal models, tadalafil increases the brain cGMP level and is associated with enhanced angiogenesis and neurogenesis [14].

In our results, all groups demonstrated histological changes in the corrugated internal elastic lamina, smooth muscle cell proliferation, and continuity of the internal elastic lamina in the basilar arteries. Desquamation of endothelial cells was observed in the SAH and tadalafil groups. In the arterioles, the internal elastic lamina in the drug-administered groups was flat compared with the SAH group. Disruption of the internal elastic lamina and loss of tight junctions have been observed in vasospasm animal models [26,29,30], but were possibly not observed in our study because the damage to the internal elastic lamina depends on the vasospasm progression. In IHC staining, the expression levels of BCL2 and Bax in the basilar arteries and of BCL2 in the arterioles were lower in the drug-administered groups than in the SAH group, but this was not statistically significant. The expression of Bax in the arterioles was significantly lower only in the nimodipine group. However, although the Bax expression was significantly lower in the tadalafil and combined groups compared with the control group, the *p* value remained significant after post hoc analysis only in the nimodipine group. In a histological comparison of the basilar artery after the administration of tadalafil and nimodipine reported by Youm et al. [13], tadalafil inhibited smooth muscle proliferation in the tunica media and maintained the internal diameter of the basilar artery, similar to nimodipine. This study included 33 experimental animals, and we believe that an increase in the number of experimental subjects in our research would provide more evidence for the efficacy of tadalafil for endothelial apoptosis. The reason why nimodipine and tadalafil have little anti-apoptotic effect in the arteries is because extrinsic factors such as oxyhemoglobin or free radicals present in the subarachnoid space make it difficult to maintain an intracellular calcium concentration, leading to a further calcium-dependent apoptotic cascade [8]. In arterioles that are less affected by extrinsic factors, intrinsic factors such as vasoactive amines (NO, endothelin, etc.) secreted by endothelial cells play a more important role, and nimodipine and tadalafil are expected to have greater effects in the arterioles. Nimodipine has a better vasodilatory effect on arterioles than on arteries [31].

Mechanistically, PDE-5 inhibitors inhibit the conversion of cGMP to GMP, induce NO formation and vasodilatation, and activate the PI3K/AKT/eNOS system [16,32,33]. The PI3K/AKT/eNOS signaling pathway comprises three subtypes of PI3Ks [34]. Class I PI3Ks are related to anti-apoptosis, cell proliferation, and anti-inflammation [35,36], class II PI3Ks are related to smooth muscle cell contracture, glucose metabolism, and extracellular secretion [37], and class III PI3Ks are related to endosome transport and autophagy [38]. This activation of the PI3K/AKT/eNOS pathway may exert anti-inflammatory, anti-apoptotic, and vasodilatory effects. We believe that PDE-5 inhibitors can prevent NO downregulation by maintaining the level of intracellular cGMP, inhibiting smooth muscle proliferation, and preventing vessel wall tonic contracture, and that activation of the PI3K/AKT/eNOS signaling pathway has anti-inflammatory, anti-apoptotic, and vasodilatory properties. This mechanism shows potential as a future treatment for DCI.

The limitations of this study areas follows: First, a quantitative analysis such as Western blot was not performed. Quantitative analysis is thought to be able to explain the results of a study more clearly, but our institution did not support a quantitative analysis. Second, the limited number of experimental animals may have caused statistical errors and difficulty in generalizing the results. Third, various apoptotic proteins were not included, and these proteins may be related to the new apoptotic mechanism between SAH and endothelial apoptosis. There are still some problems facing the clinical application of tadalafil, and additional research is needed to prove its benefits. Further work could also include conducting a quantitative study on other endothelial apoptosis-related proteins besides BCL2 and Bax. In addition, regarding the PI3K/AKT/eNOS pathway, it is necessary to track each by-product through the three PI3K pathways after tadalafil administration. Additionally, the co-administration of nimodipine and tadalafil did not produce a synergic effect with respect to apoptosis, so future studies on drug interactions are needed.

## 5. Conclusions

Tadalafil, which has the longest half-life among PDE-5 inhibitors, does not prevent endothelial apoptosis as much as nimodipine, but it still prevents smooth muscle cell proliferation in the tunica media, restores damaged endothelial integrity, increases cerebral blood flow, and is thought to have various neuroprotective functions through the PI3K/AKT/eNOS signaling pathway. We believe that more in-depth research on tadalafil in the future will prove the therapeutic potential of this drug for DCI.

## Figures and Tables

**Table 1:** Comparative analysis of immunohistochemical staining (BCl2 vs. Bax) scores of each group of basilar arteries and penetrated arterioles.

Group (n)		Bcl2 Staining Score (Average ± SD)	*p* ^†^	Bax Staining Score(Average ± SD)	*p* ^†^	Groupwise Comparison		
						Group	*p* ^‡^	*p* ^§^
Basilar artery	C (15)	1.07 ± 1.03	<0.001	0	<0.001	C vs. S	<0.001	<0.001
	S (13)	4.77 ± 1.17		3.31 ± 1.49		C vs. N	0.002	0.001
	N (14)	3.57 ± 1.40		2.43 ± 1. 65		S vs. N	0.311	0.720
Penetrated arteriole	C (19)	1.70 ± 0.95	<0.001	0	<0.001	C vs. S	<0.001	<0.001
	S (24)	4.65 ± 1.27		4.00 ± 2.41		C vs. N	0.001	1.000
	N (21)	4.15 ± 1.40		1.40 ± 1.39		S vs. N	0.724	<0.001

C: control group; S: SAH–saline group; N: SAH–nimodipine group. *p* ^†^ value according to Kruskal–Wallis test. Each groupwise comparison was analyzed using a Kruskal–Wallis test and adjusted with Bonferroni correction. *p* ^‡^ is the value of Bcl2 and *p* ^§^ is the value of Bax. SAH, subarachnoid hemorrhage.

## Data Availability

The data presented in this article are available upon request to the corresponding author.
